# Yersiniosis and Food Safety

**DOI:** 10.1155/2012/605037

**Published:** 2012-03-20

**Authors:** Latiful Bari, Dike O. Ukuku, Kenji Isshiki, Ramesh C. Ray, Didier Montet

**Affiliations:** ^1^Food Analysis and Research Laboratory, Centre for Advanced Research in Sciences, University of Dhaka, Dhaka 1000, Bangladesh; ^2^Food Safety and Intervention Technologies, USDA-ERRC-ARS, Wyndmoor, PA 19038-8598, USA; ^3^Food Chain Safety and Quality Management Laboratory, Division of Marine Life Science, Faculty of Fisheries Sciences, Hokkaido University, 3-1-1 Minato-cho, Hokkaido Hakodate, 041-8611, Japan; ^4^Regional Centre, Central Tuber Crops Research Institute, Orissa Bhubaneswar 751019, India; ^5^Cirad, UMR 95, 73 rue Jean-François Breton, 34398 Montpellier Cedex 5, France

This special issue of Journal of Pathogens was designed to share some of the interested scientific studies published on yersiniosis, a foodborne outbreaks associated with consumption of food contaminated with *Yersinia*. In this issue, the focus was on yersiniosis-related foodborne illnesses, behavior of *Yersinia *in foods, incidence, persistence, survival, or growth, outbreaks and surveillance, zoonosis virulence and pathogenesis, detection/identification, mechanisms to grow in foods, and public health. *Yersinia *belongs to the *Enterobacteriaceae *family and is often isolated from clinical specimens. Three *Yersinia *strains, namely, *Y. enterocolitica, Y. pseudotuberculosis, *and *Y. pestis, *are pathogenic to humans and are widespread among various animal species and in the environment. They are transmitted to humans by the oral route and cause intestinal symptoms such as abdominal pain, diarrhea, and fever. These species are found all over the world, with a higher incidence in temperate and cold environments. 

In this special issue, behavior of *Yersinia enterocolitica *in foods, their incidences, possible route of contamination, persistence, factors that influence the survival, or growth in food, soil, and water are reviewed by Bari et al. 

The epidemiology, outbreaks and surveillance, and zoonosis of *Yersinia *spp. and their current status in different foods and environments are discussed by A. Rahman et al. 

The molecular insight of virulence of *Yersinia enterocolitica*, mode of transmission of virulence, and their factors are covered by Y. Sabina et al. 

The pathogenesis of *Yersinia enterocolitica *and *Y. pseudotuberculosis *in human yersiniosis, their genomics, mechanisms of infection, and host responses including the current state of surveillance, detection, and prevention of yersiniosis, are presented by C. L. Galindo et al. 

The virulence plasmid (pYV) associated with the expression of phenotypic virulent in pathogenic *Yersinia *species and procedure to monitor the presence of virulence plasmid in *Y. Pestis *during storage and a convenient culture method for monitoring the presence of virulent plasmid in food are discussed by S. Bhaduri and J. L. Smith. 

A highly sensitive, specific, and accurate selective chromogenic culture plate method that has been developed for detecting pathogenic *Y. enterocolitica *from pig tonsils was discussed by M. Denis et al. 

H. Fukushima et al. reviewed and discussed the commercially available conventional and PCR-based procedures for specific detection of pathogenic *Y. enterocolitica *and *Y. pseudotuberculosis *in foods. 

J. Gui and I. R. Patel reviewed and discussed the recent advances in molecular technologies and their application in detecting pathogenic *Yersinia *in foods. 

R. Das et al. reported in their research article the presence of a novel single-stranded DNA in *Yersinia frederiksenii *and their genomic analysis, and they found that enzyme might be responsible for the transposition of this novel retron element. 

In the last reviewed article, S. N. Aziz and K. M. S. Aziz discussed the theoretical modeling to avoid exposure of *Yersinia enterocolitica *infections in foods.

*Latiful Bari* *Latiful Bari* *Dike O. Ukuku* *Dike O. Ukuku* *Kenji Isshiki* *Kenji Isshiki* *Ramesh C. Ray* *Ramesh C. Ray* *Didier Montet* *Didier Montet*

